# Diet and Diet Fads

**Published:** 1908-11-14

**Authors:** 


### Dietetics.

### DIET AND DIET FADS.

There is perhaps no subject upon which the practitioner finds it so difficult to lay down the law to his patients as on the question of diet. In acute illnesses, when the necessity for imperative obedience to the doctor's orders is obvious to the patient and friends as well, it is comparatively easy to prescribe a rigid diet, and to enforce it. Few patients, when labouring under several degrees of fever and prostrated by an attack of influenza or some other acute condition, have the strength, even if they have the desire, to resist the limitations which are imposed upon them in the matter of eating and drinking. In such circumstances the doctor has an easy task: he has only to negative this or that, and usually the patient's friends fall in readily with his wishes. Far different, however, is the case in the stages of convalescence, or in those conditions where a chronic lesion demands constant care and attention to the diet scale. The popular notion is that convalescents must be fed up. Stout and steak, in the opinion of that much-enduring individual the man in the street, make blood, and no medical man wins in popularity by diminishing the intake of such succulent morsels or such strengthening decoctions as the friends may press on the recovering patient. The doctor who "starves" a patient is not kindly regarded, even though he may point to the practical results of his methods, and notwithstanding the fact that patients have experienced the benefit in their individual cases of a little judicious fasting on occasion. No popular error is so widespread or so deeply rooted as the common notion that the benefit a man receives from his food is directly proportionate to the quantity he eats. It is an error diet reformers, both medical and lay, have been at pains to point out from the days of Sathrusta to the present time, and there is every likelihood that they will have to continue to point it out for very many decades to come. In the medical Vedas it is clearly stated "fasting hurts no man, but a full stomach engenders evil humours," and still more clearly has the impresario of the well-known Alexis St. Martin, Dr. Beaumont, enunciated the error of the popular fallacy when he wrote: "The system requires much less than is generally supplied to it. . . . If more be taken than the actual wants of the economy require, the residue remains in the stomach, and becomes a source of irritation, produces a consequent aberration of function, or passes into the bowel in an undigested state. . . . Dyspepsia is oftener the effect of over-eating and over-drinking than of any other cause." These are facts that every medical man would do well to impress upon his patients, and if the education of the community in matters of diet were systematically done by family practitioners, less would be heard about diet fads and faddists, and the path of the doctor in dealing with convalescent patients would be made very much more smooth than it is at present.

Annoying and irritating as are the over-feeders—the subjects who object to prescriptions for limiting the diet, and who maintain that "a patient must eat to live," often carrying their principles so far, however, as to make it evident that they desire to invert them—the diet faddist is a still more annoying and irritating being. Of late years his class has multiplied out of all proportion to the benefits he confers on the race. Formerly he ramped alone, like the lion on the shield of Scotland; but now he is banded into societies, and may aptly have the adjective gregarious applied to him. We all of us have met with him in various modifications. There is the man who zealously avoids carbohydrate food because his father died from diabetes; the man who takes no asparagus because it contains "purin bodies"; and the man who insists upon having nuts even with his afternoon tea. Against this strong and increasing band the practitioner, if he is a sensible, level-headed being, who is not himself led away by one or other pet fad, will inevitably collide in the course of his career. The collision may be slight, and occasion nothing but a smile or a mild battle of words, or it may, as unfortunately happens now and then, be of graver importance and lead to the loss of a patient. The faddist does not want to be told his errors: he dislikes nothing so much as "being made a fool of." It is useless to quote authorities to him: he will retort by quoting opposites. Usually he has a smattering of science, just as the "authorities" who write for his special delectation have gained a certain acquaintance with physiological and biological theories, and he is an expert at misapplications. For all that, he is an opponent to be reckoned with, and the practitioner should feel more grateful for one faddist converted than for half a dozen over-feeders induced to see things connected with diet in a reasonable light. In dealing with diet and diet fads, it is highly necessary that the practitioner should have a definite opinion on the subjects he discusses without being in any way a bigot or a blind adherent to any one school. It is fortunate, indeed, that the majority are such, but there is a deplorable tendency towards faddism in matters dietetic, even in the profession. There is the school that taboos alcohol, the section that raves against meat, and the regiment that sees disease in other articles of diet. These medical faddists are not numerous, but they exist, and their very existence is a staff and support for the lay faddist. We have no desire to discuss them here. In ensuing articles we hope to deal with their fads, in so far as these concern general dietetics. For the present we will content ourselves by pointing out that science is gradually coming back to a distinctly anti-faddist level. We are reacting in favour of common sense. It is becoming recognised by physiologists that desire and appetite are not pathological curiosities, and that where a taste exists the onus of proof is on the physician to show that indulging would be detrimental to the organism. In matters dietetic, scientific regulation is a grand thing, but it should go hand in hand with common sense and a careful study of the temperament and tastes of the individual. No one can presume to lay down rules that should regulate the diet of a patient until he has profoundly studied the influence of various articles of diet upon the health of such patient—until, in fact, he knows the patient's indiosyncrasies. Ordinarily such a profound study is entirely out of the question; life is too short and the average practitioner is far too busy for it. He must, therefore, content himself with the patient's own evidence, and comfort himself with the reflection that "if a man at forty has not found out what will agree with him and what disagree, no doctor in the world can make him any wiser than he is." The statement may not be absolutely true but it has a germ of truth in it, and it draws attention at least to a point that no practitioner who is interested in matters of diet and dietetics generally, should ever overlook—namely, that generalisations are unwise and that in medicine a cut-and-dried regimen is not always the best.

